# Social Participation and HRQoL with Neurodevelopmental Disabilities: Mediation by Everyday Living Capabilities

**DOI:** 10.1093/geroni/igaf122.4199

**Published:** 2025-12-31

**Authors:** Rongfang Zhan, Kayi Ntinda Ntinda, Elias Mpofu

**Affiliations:** University of North Texas, Denton, Texas, United States; UT health Rio Grande valley, Edinburg, Texas, United States; University of North Texas, Denton, Texas, United States

**Keywords:** Social Participation, neurodevelopmental disabilities, health-related quality of life, mediation analysis

## Abstract

**Background and aims:**

Social participation is a key determinant of health-related quality of life (HRQoL) of adults with neurodevelopmental disabilities (NDDs), yet their social participation for HRQoL in everyday living remains unknown. This study investigated and social participation HRQoL relationship, with everyday living capabilities as a mediator.

**Method:**

The of 304 adults with NDDs (females = 83.3%; age range 18 to 69 years old). completed standardized social participation, Health related quality of life (HRQoL) and everyday capabilities measures. Multi-group path analysis was employed to examine self-perceived everyday living capabilities meditation of the association between social participation and health-related quality of life (HRQoL) across the two groups.

**Results:**

For adult autistics, both the indirect effect (β = .170, 95% CI [.071, .179]) and the direct effect of social participation on HRQoL (β = .177, 95% CI [.040, .221]) were significant, indicating partial mediation of capabilities. For adults with intellectual disability, the direct effect of social participation on HRQoL was nonsignificant (β = .174, 95% CI [–.012, .224]), indicating full mediation.

**Conclusion and implications:**

These results underscore the importance of personal factors in the HRQoL of adults with NDDs. By implication, policy and program development initiatives should improve everyday living needs for the improved quality of life for this population.

